# Supplementary material for: Mechanism of Charge Transport in Lithium Thiophosphate
Source: Chem Mater. 2024 Feb 5;36(3):1482–96. doi: 10.1021/acs.chemmater.3c02726 (PMC10870718; doi:10.1021/acs.chemmater.3c02726)
Supplement: Supplementary file 1 — cm3c02726_si_001.pdf [file cm3c02726_si_001.pdf]

Mechanism of charge transport in lithium thiophosphate
Supporting Information

Lorenzo Gigli,[1,†] Davide Tisi,[1,†] Federico Grasselli,[1] and Michele Ceriotti[1,*]
[1]*Laboratory of Computational Science and Modeling, Institut des Matériaux, École Polytechnique Fédérale de Lausanne, 1015 Lausanne, Switzerland*
(Dated: January 5, 2024)

† These authors equally contributed to this work
* michele.ceriotti@epfl.ch

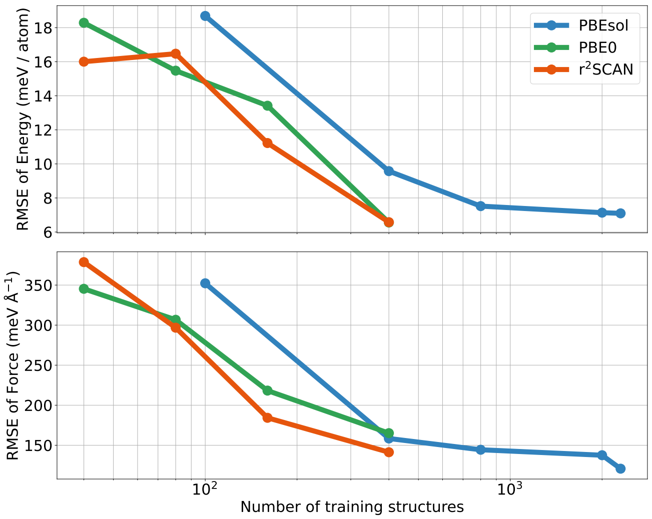

FIG. S1. Learning curves for all the potentials.

I. ML MODELS

We construct the MLPs using the well-established SOAP-GAP approach [1] to fit energies E and atomic force components $\{\mathbf{f}_l\}_{l=1}^{N}$, where N is the number of atoms, by means of Kernel Ridge Regression. In other words, we write the target properties as a sum of local, atom-centered, contributions:

$$E = \sum_{i\in A} E_i(A_i) = \sum_{i\in A}\sum_{j\in S} w_j K(A_i, S_j) \tag{S1}$$

$$\mathbf{f}_l = -\boldsymbol{\nabla}_l E \tag{S2}$$

In this equation, A_i represents the environment surrounding atom i and $E_i(A_i)$ the energy associated with it. Each of these local energies is then expanded as a sum of kernel contributions $K(A_i, S_j)$ that measure the correlation between the environment A_i and a set of representative environments S_j, the so-called sparse set S. The definition of the sparse set from the original training set is a necessary step in the ML regression procedure as it controls the computational cost of calculating energies and forces and sets a boundary to the number of kernel contributions that need to be computed at the fitting step. The kernel $K(A_i, S_j)$ needs to be computed using a set of structural fingerprints and an explicit form of the kernel function K. In this paper, we use the SOAP power spectrum features $\mathbf{p}$ as described in Bartók *et al.* [1] and K is a polynomial kernel:

$$K(A_i, S_j) = (\mathbf{p}_i \cdot \mathbf{p}_j)^{\zeta} \tag{S3}$$

where $\zeta = 2$. Finally, the weights w_j related to each atomic environment are determined by ridge regression. Further details on how to appropriately construct MLPs with the SOAP-GAP method can be found in [2].

We use this approach to fit a reliable potential at the PBEsol level (ML-PBEsol). Since the training sets computed with r^{2}SCAN and PBE0 are smaller due to their larger computational complexity, we find it beneficial to construct the corresponding MLPs by using the ML-PBEsol model as a baseline and fit an accurate model for the target functional using a two-level machine learning scheme [3]. This means that we fit a new model using Eq. (S1), where the target energies and forces now represent the deviations between ML-PBEsol and the r^{2}SCAN/PBE0 target. We then add this additional contribution to the baseline model to obtain the final model. In this way, we manage to leverage the accuracy of the baseline model to the more expensive reference and find that the resulting model is more accurate than the direct model.

All the ML models are obtained using LIBRASCAL [4] and they share the same hyperparameters, shown in Table S1. In addition, the atomic environment is defined by a smooth cutoff function with a long-algebraic decay of the form:

$$u(r) = \frac{c}{c + (r/r_0)^m} \tag{S4}$$

with $c = 1$, $r_0 = 3.5$ Å and $m = 4$. All the models are constructed with both feature and sample sparsification computed using the Farthest Point Sampling implemented in `skmatter` [5]. The models use a total of 600 sparse features, 4500 sparse environments for the GAP-PBEsol model and 5000 sparse environments for the other two delta models. Fig. S1 shows the behaviour of the root mean square error (RMSE) of energy and forces for all the models as function of the number of training structures.

interaction cutoff	5 Å
n_{max}	8
l_{max}	6
Gaussian smearing	0.3 Å
radial basis	Gaussian Type Orbitals

TABLE S1. Parameters of the SOAP-GAP fits used in LIBRASCAL [6]

II. KERNEL PRINCIPAL COMPONENT ANALYSIS

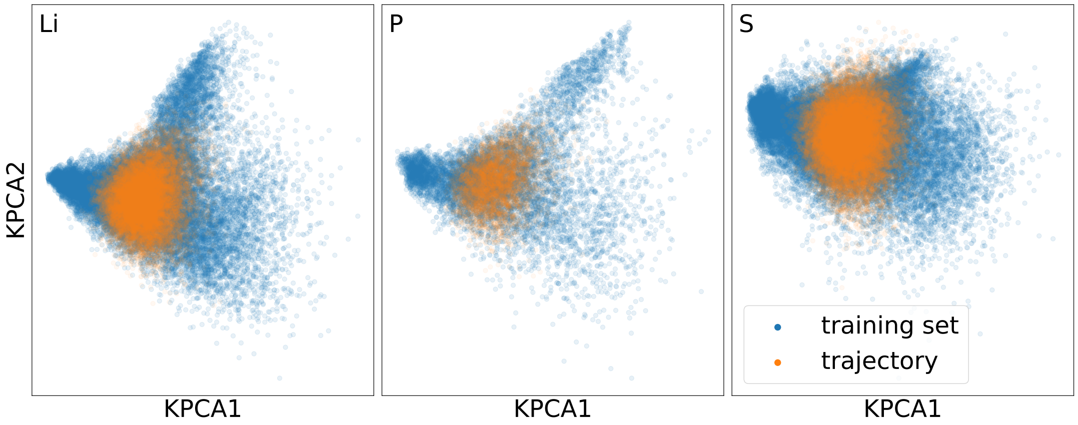

FIG. S2. KPCA per species of the local environments in the training dataset (blue) and the projection of the environments of a trajectory computed with the ML-PBEsol at 700K (orange), where rotations of PS_4 tetrahedra are present, on the training dataset KPCA map. The first two principal component, which account for 70% and 14% of the total variance of the dataset environments' feature vectors, are displayed.

In our work, we showed that the conductivity of Li_3PS_4 is largely affected by the rotation of the PS_4 tetrahedra, during the dynamics. These events are generally rare, particularly at low temperatures, but given their relevance, it is essential that they are well-represented in our training set. To show that this is indeed the case, we perform a (sparse) kernel principal component analysis (KPCA) [7] of the local environments of each atomic species in the training dataset, and we project the local environments of the snapshots of a trajectory where these rotations are present on the KPCA map of the training set. Fig. S2 contains, for each species, the first two principal components (which explain 84% of the total variance) for environments in the dataset (blue) and in a trajectory with ML-PBEsol at 700 K (orange). One can see that all the environments from the trajectory are fully contained in the region defined by the environments from the training dataset. The authors are aware that a projection into two principal components, like in Fig. S2, might not be a complete assessment of the robustness and accuracy of the training set [8]. In order to give further evidence that our dataset well describes the complex environments that occur during the rotation of the tetrahedra, in the next section we compute the local prediction rigidity (LPR) introduced in Ref. [9].

III. LOCAL PREDICTION RIGIDITY

The LPR is a quantitative metric of how robust the locally decomposed predictions of ML models are. It essentially captures how prone to changes (or conversely, "rigid") a given local prediction is under perturbation of the model,

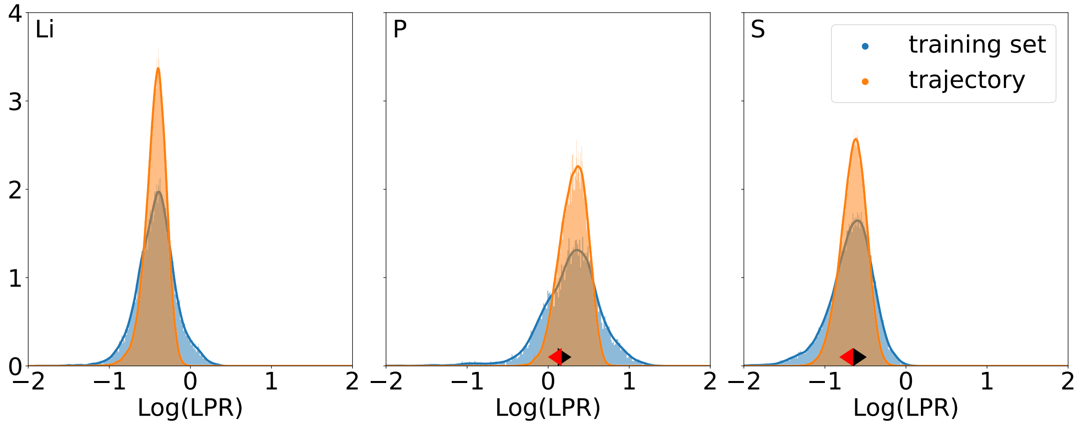

FIG. S3. Histogram of local environments and their local prediction rigidity (LPR) values expressed in base 10 logarithm, for environments contained in the training set (blue) and in a trajectory computed with the ML-PBEsol at 700K (orange), where rotations of PS_4 tetrahedra are present. Each panel corresponds to environment of a different atomic species. The triangle-shaped markers correspond to the environments of P (or S) that is included in a tetrahedra that rotates. Black right-pointing triangles correspond to environments before the rotation, while red left-pointing triangles correspond to an environment after the rotation.

given a dataset. High values of LPR imply that a prediction is robust and thus, such environment is well described by our training dataset, in principle because it is similar to many other environments therein. Fig. S3 shows the histograms of the logarithm of the LPR for the environments in the training set (blue) and for environments in a piece of a trajectory of γ Li_3PS_4 computed with the ML-PBEsol at 700K (orange), where there are rotations of the PS_4 tetrahedra. For any environment in the trajectory, the LPR is never particularly low and always lies within the range of LPR for dataset's environments. Moreover, we focus on the environments of a P (or S) atom included in a PS_4 tethrahedra that rotates during the transition. Black right-pointing triangles represent environments in a snapshot before the rotation, while red left-pointing triangles represent environments after the flip. Both kinds of environments have a relatively large LPR and are, in particular, perfectly included in the distribution of those coming from the training set, showing that they are well represented inside our dataset.

IV. MEAN SQUARE DISPLACEMENT: COMPARISON WITH AN AB INITIO CALCULATION

To show that our ML models can correctly describe dynamical properties of Li_3PS_4, we perform a 40-ps long Born-Oppenheimer *ab initio* NVT simulation at the PBEsol level for a system of 64 atoms in the α phase and 750 K. Then, we compute the mean square displacement (MSD) of Li atoms, and we compare the results with those from an equivalent NVT simulation run for 900 ps with the ML-GAP potential on the same simulation cell. Figure S4 shows that both the MSD and the diffusivity obtained from our ML model are in agreement with those obtained from the *ab initio* simulations.

V. DFT COMPUTATIONAL DETAILS

The three datasets are computed at different levels of theory. The PBEsol dataset is computed with the Quantum ESPRESSO suite of codes [10–13] with PBEsol functional [14], with a energy cutoff equal to 120 Ry. The SCAN and PBE0 calculations are computed with VASP (Vienna Ab initio Simulation Package) [15–17], taking advantage of the Projector-Augmented Wave (PAW) pseudopotentials [18, 19] provided by VASP in combination with dense k-point meshes to ensure systematic convergence of all self-consistent field (SCF) steps. Specifically, we cautiously set the kinetic-energy cutoff to 36.74 Ry (500 eV) for all functionals. Moreover, the PBE0 calculations were computed, in VASP, in two steps in order to speed up convergence: 1. First we run a SCF calculation at PBE level, with the same energy cutoff; 2. Secondly, we run the SCF calculation at the PBE0 level using the wavefunction from the previous calculation as starting point. This initialization significantly reduces the computational cost. All the DFT calculations of this work are converged within a SCF energy tolerance of 10^{-6} eV and well below the ML energy errors shown in the main text.

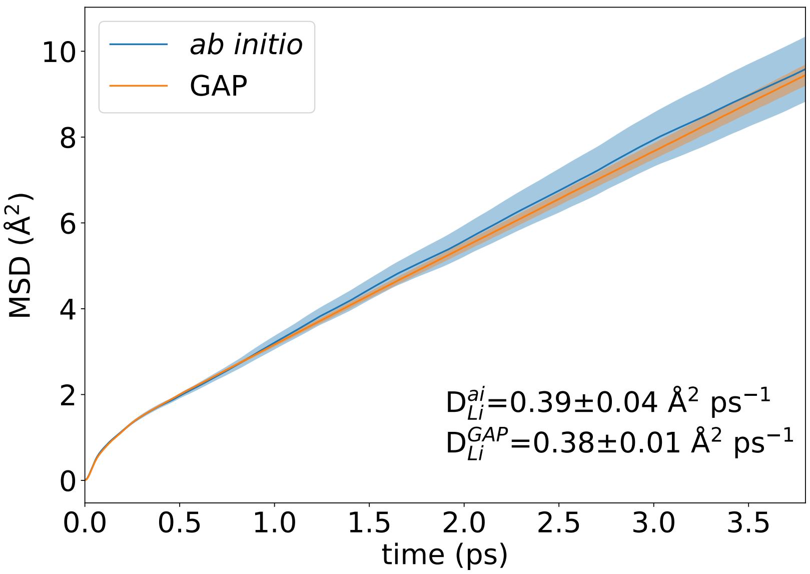

FIG. S4. Mean square displacement of the Li atoms obtained from simulations for a system of 64 atoms of Li_3PS_4 in the α phase at 750 K. The blue line is the result of a Born-Oppenheimer *ab initio* simulation at the PBEsol level; the orange line, instead, is obtained via the PBEsol-GAP potential. The shaded areas represent the uncertainty from standard block analysis.

Car–Parrinello (CP) Molecular dynamics in the NVT ensemble [20] were performed to generate a preliminary dataset of Li_3PS_4 structures in the β and γ polymorphs by using the CP module of Quantum ESPRESSO. The simulations were carried out at 250, 500 and 1000 K for the two phases and with three different reference volumes: 4100, 4450 and 4800 a.u.^{3} for the β structure with 32 atoms and 2000, 2100 and 2230 a.u.^{3} for the γ structure with 16 atoms. A fictitious electronic mass of 200 a.u. and a time step of 4 a.u. (ca. 0.09 fs) were applied. The electronic and ionic motions were propagated by a standard Verlet algorithm. The ionic and cell temperatures were controlled by a Nosé thermostat [21]. Periodic boundary conditions were applied. The CPMD simulations were performed at Γ using the PBEsol [14] with RRKJ ultrasoft pseudopotentials [22]. The energy cutoff of the plane wave basis set was set to 40 Ry, while the energy cutoff for the charge density was set to 320 Ry.

VI. BAND STRUCTURE OF THE γ-PHASE

Figure S5 shows the band structure and the electronic density of states of the γ phase. As for the β phase, the PBE0 predicts the largest band gap.

VII. HEAT-QUENCH TRAJECTORY

The simulation protocol works as follows: the simulation starts from a γ structure at 500 K and the temperature is linearly increased to 800 K in 250 ps. We then let the system equilibrate at 800 K for a 250 ps time lapse, after which the temperature is decreased back to 500 K in 250 ps. The system is finally equilibrated again at 500 K. Figure S6 shows the dynamics of the two collective variables defined in Sec. IIB of the main text during the heat-quench simulation, showing an hysteresis behaviour, where the mixed β and α phase persists after cooling. Figure S7 show the temperature profile of the heat-quench protocol.

It is important to note that the onset of this transition is due to thermal activation of the rotational degrees of freedom of the tetrahedra, and for temperatures $T < T_c$ no phase transition ever occurs in any of the MD simulations. This is a result of the simulations not being ergodic, as evidenced by the fact that at low temperatures only small PS_4 angular fluctuations occur and there is no signature of any global reorientation of the tetrahedra. This is a limitation, as the lack of ergodicity of the MD does not allow us to provide an estimate of the relative thermodynamic stability of the different polymorphs. For this reason, we cannot provide a direct comparison with experiment of our estimated transition temperature from the γ to the disordered β/α structure. In order to ensure however that the phase transitions observed in the main paper are a physical feature of this material, we run a heat-quench simulation with fully-flexible cell up to 800 K (see Supplementary Information) and qualitatively observe the onset of the same

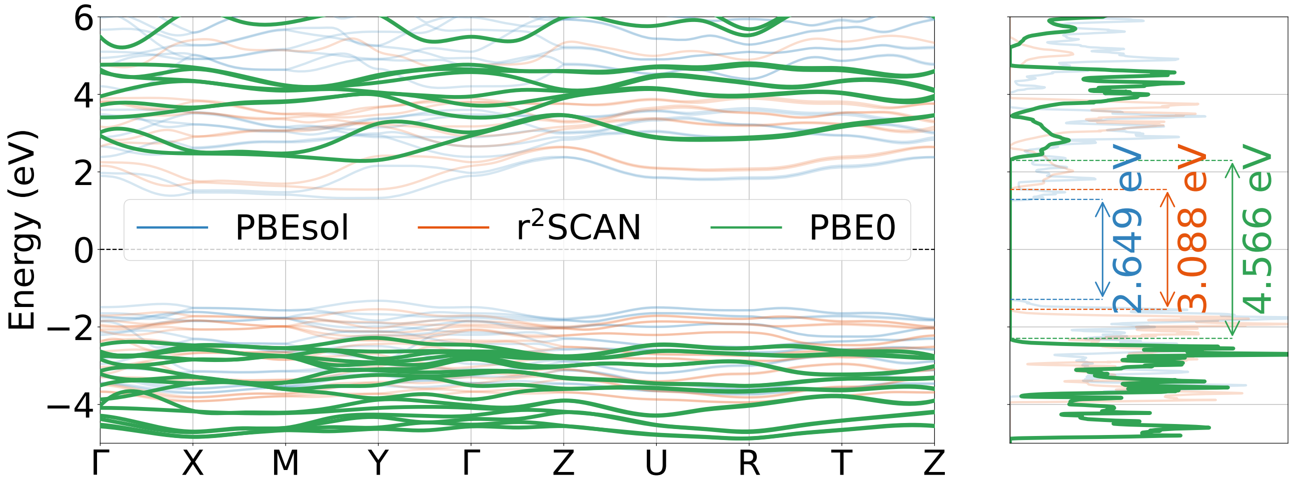

FIG. S5. Electronic bands and density of states of Li_3PS_4 for the γ phase.

phase transition, with a strong hysteresis of the CVs upon cooling the system back to 200 K. Consequently, the use of the NpT ensemble is justified, as we ensure that the system can sample configurations within the range of stability of each phase, while it is still free to undergo phase transitions when the rotations of the PS_4 groups are thermally activated.

VIII. COLLECTIVE PLANE FLIPPING IN LI_3PS_4

In Section IIIB of the main text, we show how we can study the collective variables dynamics during a simulation to mark the phase transition. In the main text, by analysing the microscopic mechanism of this transition, we saw how it is characterized by a collective flip of PS_4 the tetrahedra of a specific plane. Figure S8 shows, for a simulation at the PBE0 level and initialized in γ, the evolution of each s^{C}, the order parameter that measures the average alignment of PS_4 tetrahedra for *each* (010) plane, $\mathrm{C} = 1, \ldots, 6$. This analysis highlights the presence of discrete jumps that correspond to the collective flip of entire [100] rows of PS_4 tetrahedra across each plane, similar to what happens in Fig. 5 in the main text.

IX. MELTING

The effects of melting on the Li_3PS_4structure can be seen from the P-P and P-S radial distribution functions, $g_{\mathrm{PP}}(r)$ and $g_{\mathrm{PS}}(r)$. Fig. S9 shows that, while increasing the temperature, the sharp peaks in g_{PP}, typical of solid behaviour, become broader and broader, and at 900K the behavior is more typical of a liquid. On the other hand, the first two peaks of g_{PS} remain present and sharp at any temperature, showing that while the solid matrix melts into a "liquid-like" phase, the PS_4 tetrahedral units are preserved.

X. ELECTRICAL CONDUCTIVITY

The negative slope of the profiles of σ with respect to the inverse temperature (Fig. 8 in the main paper) is typical of the Arrhenius plots. In fact, since lithium-ion diffusion occurs via thermal activation, we can mathematically relate the Nernst-Einstein conductivity σ_{NE} with the activation energy of the Li-hopping process:

$$\sigma_{\mathrm{NE}} T = \sigma_{\mathrm{NE}}^0 T^0 \exp\left(-\frac{E_A}{k_B T}\right) \tag{S5}$$

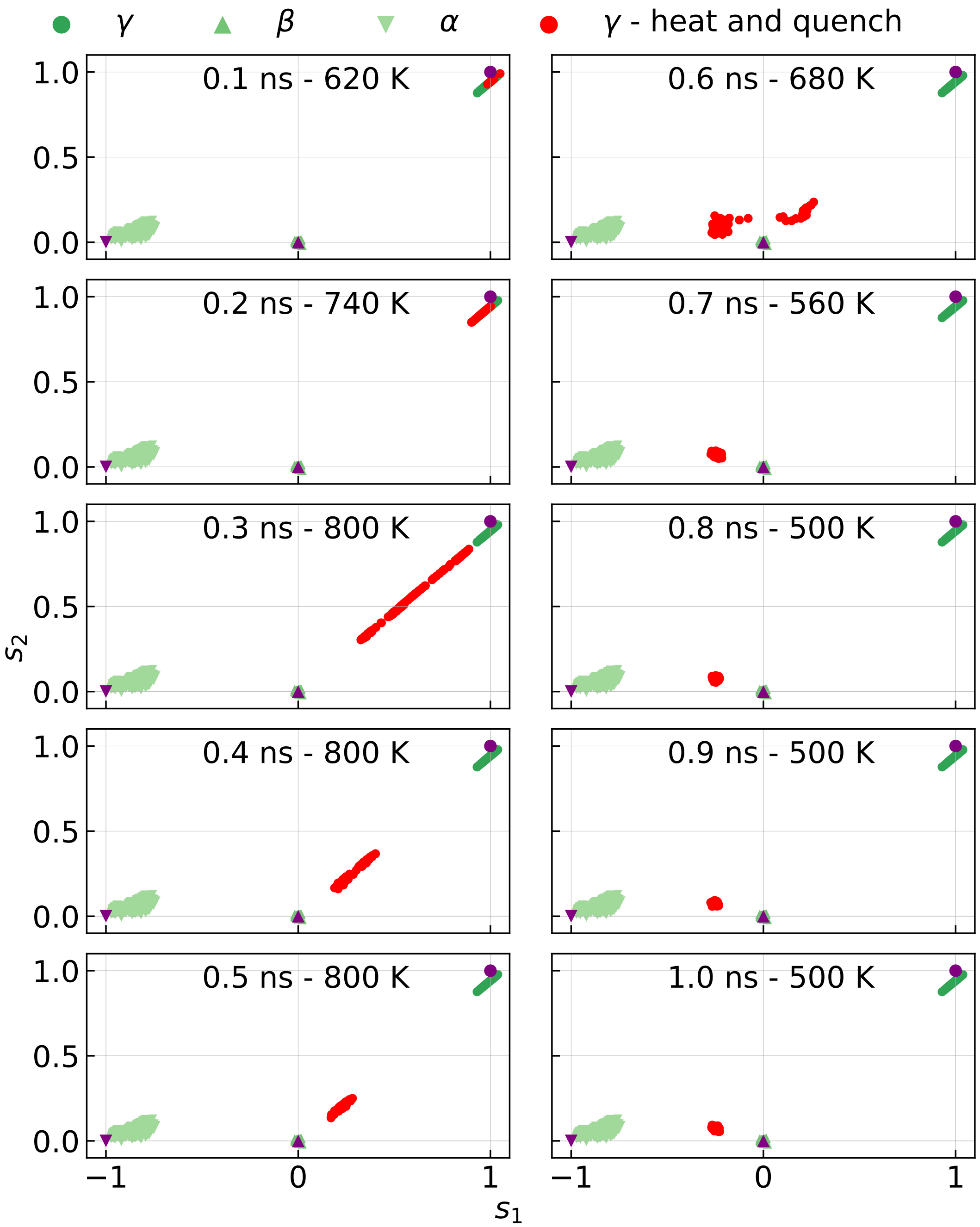

FIG. S6. Evolution of the collective variables (CVs) along a heat-quench trajectory starting from the γ structure. A sample of values of the CVs for γ structures generated from NpT simulations with $T < 750K$ and β and α structures are provided as reference. The evolution of the CV clearly shows substantial hysteresis and the structure that emerges from the quench is the same structure with mixed β and α alignments discussed in Sec. IIIC of the main paper. The purple symbols correspond to the values of the CVs for the ideal α, β and γ structures.

In Fig. S10 we show σ_{NE} as predicted by the ML models for simulations initialized in the three phases. We also plot the fits to the Arrhenius equation of Eq. (S5) (black solid lines) that yield the activation energies listed in Tables III and IV in the main paper. For the simulations initialized in the γ structure, we fit the curve both below and above the transition point discussed in Sec. IIIC. In Fig. S11, we compare the GK and NE conductivities for simulations in the α phase and the extrapolation of σ_{GK} to room temperature (see Table IV in the main paper). Finally, in Fig. S12 we show the mean square displacement of lithium ions as a function of time in a simulation run at 750 K with the ML-PBE0 model and initialized in the γ structure. This simulation corresponds to the PS_4 flips observed in Fig. 6 in

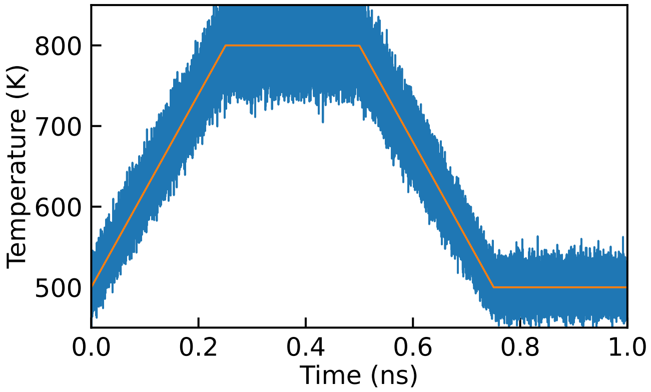

FIG. S7. Temperature profiles of a heat-quench trajectory starting from the γ structure.

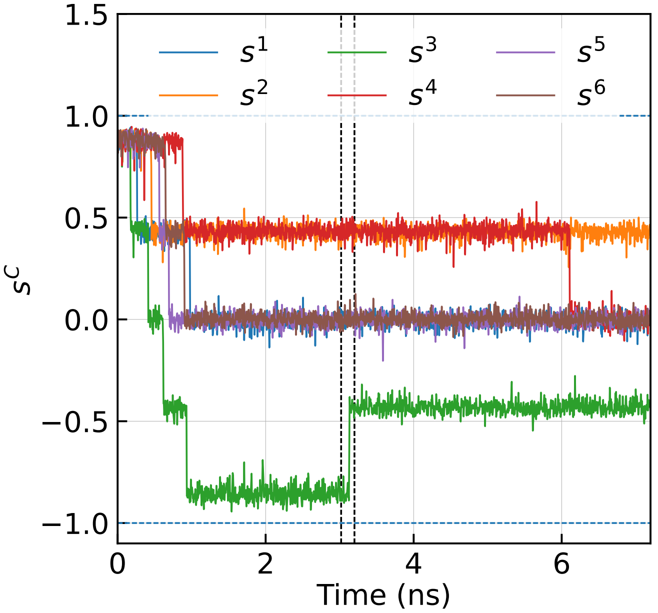

FIG. S8. Time evolution of the collective variable s^{C} defined in Sec. IIB of the main paper for each (010) plane of a NpT simulation at 750 K and run with the PBE0 functional. The dynamics of the s^{C} shows the presence of discrete jumps that correspond to the collective flip of entire [100] rows of PS_4 tetrahedra across each plane. The flips occur between the positive and negative directions of the x-axis as defined in Fig. 1 of the main paper. The plane numbers are the same of Fig. 1 of the main text. The vertical dashed lines indicate the time range of the transition shown in Fig. 5 in the main paper.

the main paper. Fig. S12 shows that the typical timescale of Li diffusion is of the order of a few ps. This time laps is much larger than the time laps between subsequent PS_4 flips along [100] rows, confirming that no paddle-wheel effect occurs. The Li diffusion coefficient that is determined through a linear fit of the line shown in Fig. S12 is equal to $(0.286 \pm 0.006)\text{Å}^2\,\mathrm{ps}^{-1}$. This value corresponds to $\sigma_{\mathrm{NE}} = (1.23 \pm 0.01)$ S cm^{-1} at 750 K (see Fig. S10).

XI. BARYCENTER AND SOLID-MATRIX REFERENCE FRAMES

A. Equivalence of formulations for total electrical conductivity

Let us consider a system characterized by one (or more) high-mobility atomic species $\mathcal{D}$ diffusing into a fixed matrix $\mathcal{M}$. In the context of Li_3PS_4, lithium ions represent $\mathcal{D}$ while the matrix of PS_4 tetrahedra is $\mathcal{M}$.

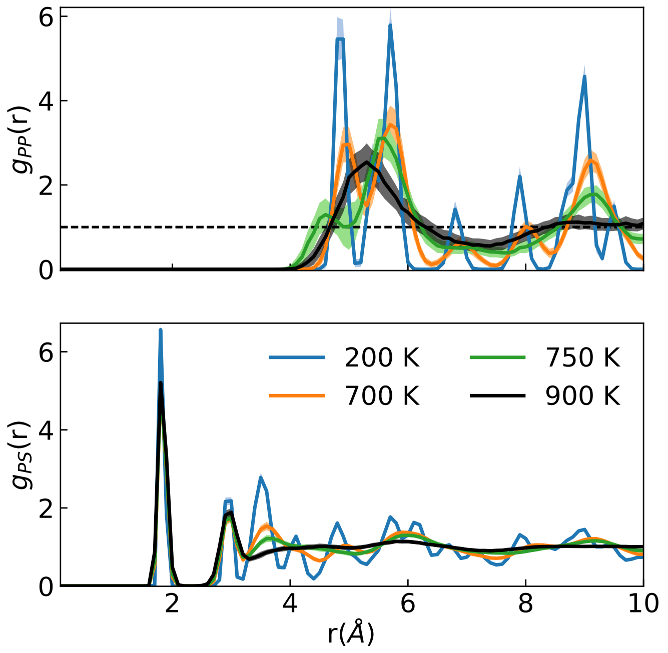

FIG. S9. Radial distribution functions of P-P and P-S atom pairs. The P-P distribution has the structure of a liquid at 900 K, indicating the melting of the solid matrix. The P-S distribution keeps a residual order at short distances at 900 K, due to the tetrahedral geometry of the PS_4^{3-} anions which is preserved also after melting.

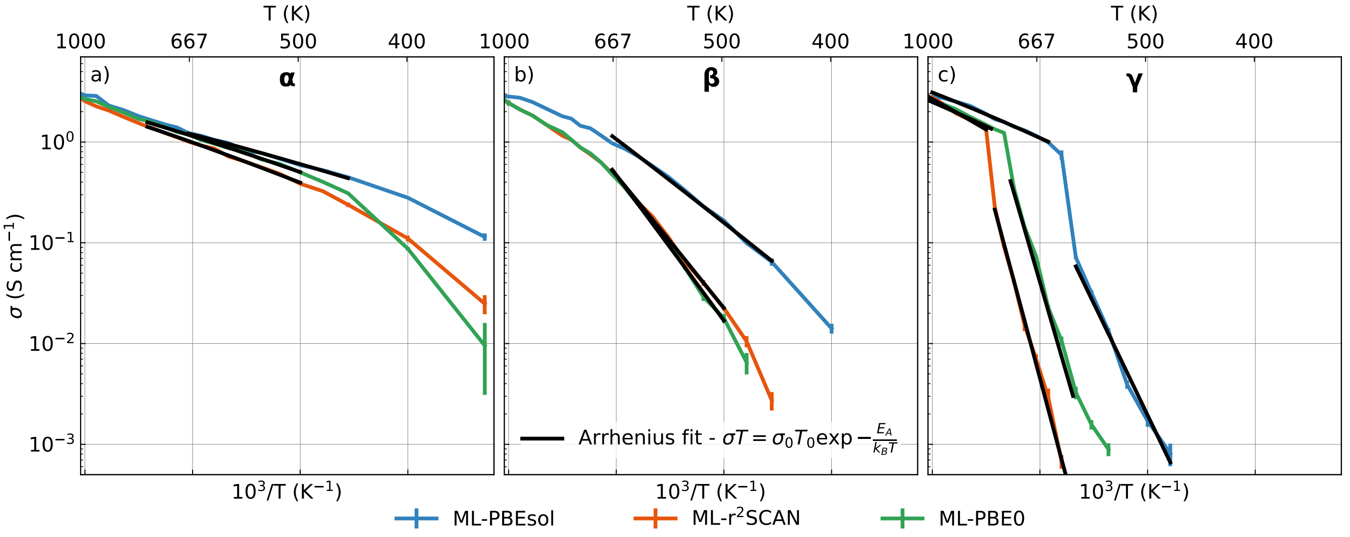

FIG. S10. Nernst-Einstein conductivities as a function of inverse temperature for simulations initialized in the α, β and γ structures, as predicted by the ML models of the main paper. The black solid lines represent the fits to the Arrhenius equation (see legend), where the fitting parameters are σ_0 and the activation energy E_{A}. For the γ profiles, we fit the conductivities both above and below the transition temperature of Sec. IIIC, to highlight the effect of the transition from γ to the β-α structure. The activation energies obtained from these fits are reported in Tables III and IV in the main paper.

In the *matrix* reference frame, only the diffusing species participate in electrical conduction. Instead, in the *barycentric* reference frame—usually adopted in equilibrium MD simulations—also the fixed matrix moves in order to compensate the net displacement of the diffusive species. This effect *must* be taken into account to avoid mistakes in the calculation of the electrical conductivity from EMD simulations [23]. Thanks to gauge invariance [24], we have some equivalent choices. One may, in fact, perform a change between the barycentric and the solid-matrix reference

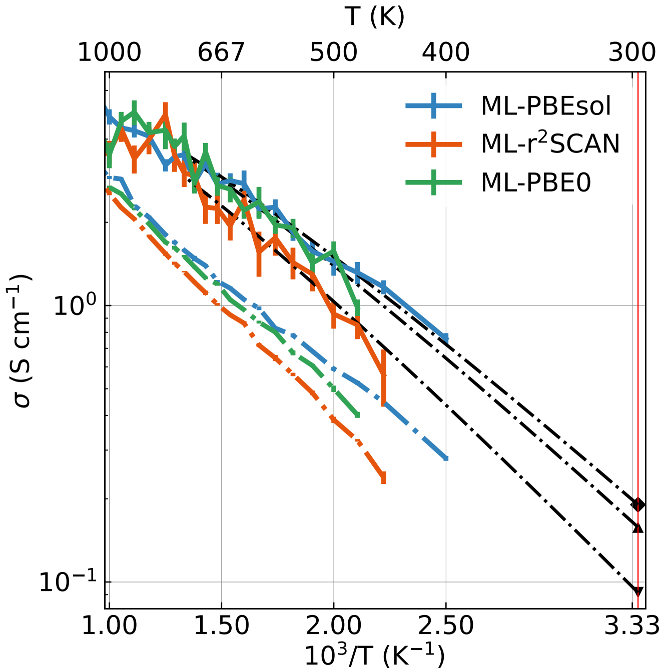

FIG. S11. Comparison between Green-Kubo and Nernst-Einstein conductivities for simulations that are initialized in the α structure. Fitting lines of the GK curves to the Arrhenius equation are shown with dash-dotted black lines. The symbols in the bottom right of the plot represent the extrapolated conductivity at room temperature (298 K), marked by a red vertical line.

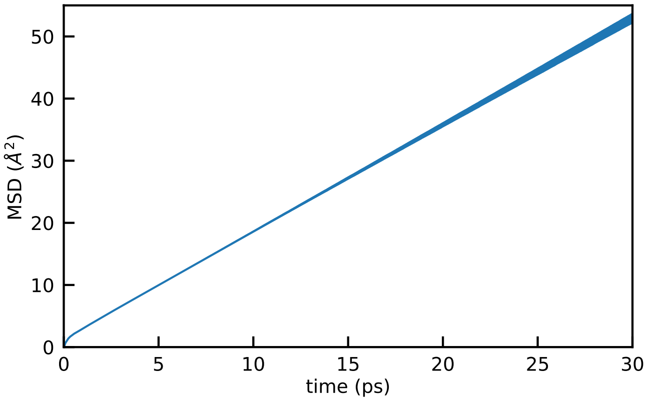

FIG. S12. Mean square displacement of lithium ions as a function of time in a simulation run at 750 K with the ML-PBE0 model and initialized in the γ structure. This simulation corresponds to the PS_4 flips observed in Fig. 6 of the main paper. The shaded area represents the uncertainty from standard block analysis.

frames, so that the atomic velocities are

$$\mathbf{v}_i^{mat} = \mathbf{v}_i^{bar} - \mathbf{w}^{bar}, \tag{S6}$$

$\mathbf{w}^{bar}$ being the velocity of the center of mass of the matrix $\mathcal{M}$ in the barycentric reference frame, and m_j the mass of atom j

$$\mathbf{w}^{bar} = \frac{\sum_{j\in\mathcal{M}} m_j \mathbf{v}_j^{bar}}{\sum_{j\in\mathcal{M}} m_j}. \tag{S7}$$

We can then employ

$$\tilde{\mathbf{J}} = \frac{1}{\Omega}\sum_{i\in\mathcal{D}} q_{S(i)}\mathbf{v}_i^{mat} \tag{S8}$$

in the GK formula for the electrical conductivity, where the sum is restricted to $\mathcal{D}$ alone, and $S(i)$ is the species of atom i. Alternatively, one may calculate directly the total flux

$$\mathbf{J} = \frac{1}{\Omega}\sum_{i\in\text{all}} q_{S(i)}\mathbf{v}_i^{bar} = \frac{1}{\Omega}\left[\sum_{i\in\text{all}} q_{S(i)}\mathbf{v}_i^{mat} + \mathbf{w}^{bar}\sum_{i\in\text{all}} q_{S(i)}\right] = \frac{1}{\Omega}\sum_{i\in\text{all}} q_{S(i)}\mathbf{v}_i^{mat}, \tag{S9}$$

where the sum is here extended to all the atoms, and where the last step follows from charge neutrality. From the gauge-invariance principle, the two choices Eqs. (S8) and (S9) are equivalent. In fact, the term $\sum_{i\in\mathcal{M}} q_{S(i)}(\mathbf{v}_i^{bar} - \mathbf{w}^{bar})$, where the sum is on atoms belonging to $\mathcal{M}$ only, is the time derivative of a bounded quantity, due to the non-diffusive behaviour of the atoms $\in \mathcal{M}$: the time-dependent *relative* displacement of any two atoms that compose the matrix (here P and S) are, in fact, bounded quantities (whenever P and S atoms do not diffuse) which do not contribute to the electrical conductivity. Instead, the use in the GK formula of the charge flux of the diffusive species

$$\mathbf{J} = \frac{1}{\Omega}\sum_{i\in\mathcal{D}} q_{S(i)}\mathbf{v}_i^{bar} \tag{S10}$$

computed in the *barycenter reference frame* leads in general to a wrong result.

B. Frame dependence of the breakdown of the electrical conductivity into species-dependent terms

In this subsection we show that, while the total electrical conductivity is reference-frame invariant (when properly computed) as shown in the previous subsection, its breakdown into species-dependent terms:

$$\sigma = \sigma_{\mathcal{D}\mathcal{D}} + 2\sigma_{\mathcal{D}\mathcal{M}} + \sigma_{\mathcal{M}\mathcal{M}}, \tag{S11}$$

where the RHS is defined thanks to GK-like equations

$$\sigma_{AB} = \frac{\Omega}{3k_BT}\int_0^\infty \langle \mathbf{J}_A(t)\cdot\mathbf{J}_B(0)\rangle\, dt, \qquad A, B = \mathcal{D} \text{ or } \mathcal{M} \tag{S12}$$

does depend on the reference frame. This is trivially verified for the fixed solid-matrix reference frame, where $\sigma_{\mathcal{M}\mathcal{M}}^{mat} = 0$ since $\mathbf{J}_{\mathcal{M}}^{mat} = \mathbf{0}$, and where $\sigma_{\mathcal{D}\mathcal{M}}^{mat}$ also vanishes, thanks to a generalization of the Cauchy-Schwarz inequality to transport coefficient [24, 25]:

$$|\sigma_{\mathcal{D}\mathcal{M}}| \le \sqrt{\sigma_{\mathcal{D}\mathcal{D}}\,\sigma_{\mathcal{M}\mathcal{M}}} \tag{S13}$$

These theoretical considerations are in fact observed in our MD simulations, as shown in Fig. S13, which displays the GK integrals as a function of the upper extremum of integration.

Finally, we show that, for a given reference frame, two different total charge fluxes leading to the same total electrical conductivity must have also the same breakdown. For simplicity, we work in the barycenter reference frame, where,

$$\mathbf{V}_{\mathcal{D}} = \sum_{i\in\mathcal{D}}\mathbf{v}_i, \qquad \mathbf{V}_{\mathcal{M}} = \sum_{i\in\mathcal{M}}\mathbf{v}_i, \qquad \mathbf{V}_{\mathcal{M}} = -\frac{m_{\mathcal{D}}}{m_{\mathcal{M}}}\mathbf{V}_{\mathcal{D}} \tag{S14}$$

(here $m_{\mathcal{D},\mathcal{M}}$ indicate molecular masses). Hence the total charge flux is

$$\mathbf{J} = \frac{1}{\Omega}\left(q_{\mathcal{D}} - q_{\mathcal{M}}\frac{m_{\mathcal{D}}}{m_{\mathcal{M}}}\right)\mathbf{V}_{\mathcal{D}} \tag{S15}$$

Let us assume that we have two different fluxes $\mathbf{J}$ and $\mathbf{J}'$, yet leading to the same conductivity, i.e.

$$\sigma = \frac{1}{3k_BT\Omega}\left(q_{\mathcal{D}} - q_{\mathcal{M}}\frac{m_{\mathcal{D}}}{m_{\mathcal{M}}}\right)^2\int_0^\infty \langle \mathbf{V}_{\mathcal{D}}(t)\cdot\mathbf{V}_{\mathcal{D}}(0)\rangle\, dt$$

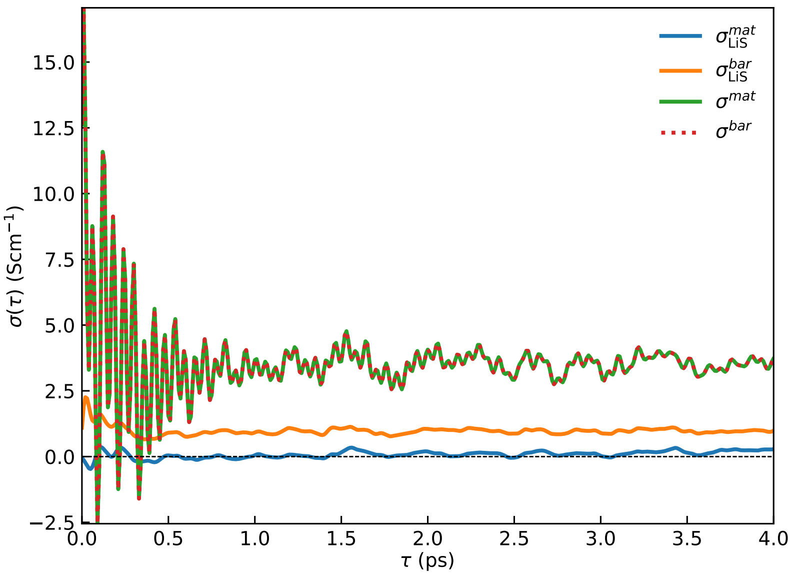

FIG. S13. Dependence of the breakdown of the electrical conductivity upon reference frame. The figure shows that the total conductivity is independent of the choice of the system of reference, while its species dependent component σ_{LiS} does. In particular $\sigma_{\mathrm{LiS}}^{mat} = 0$

$$\sigma' = \frac{1}{3k_BT\Omega}\left(q_{\mathcal{D}} - q_{\mathcal{M}}\frac{m_{\mathcal{D}}}{m_{\mathcal{M}}}\right)^2 \int_0^\infty \langle \mathbf{V}'_{\mathcal{D}}(t)\cdot\mathbf{V}'_{\mathcal{D}}(0)\rangle\, dt$$

but

$$\sigma = \sigma'$$

Then, $\mathbf{V}_{\mathcal{D}}$ and $\mathbf{V}'_{\mathcal{D}}$ differ at most by a non diffusive term $\mathbf{Q}$:

$$\mathbf{V}'_{\mathcal{D}} = \mathbf{V}_{\mathcal{D}} + \mathbf{Q} \tag{S16}$$

Therefore, $\mathbf{V}'_{\mathcal{M}} = -\frac{m_{\mathcal{D}}}{m_{\mathcal{M}}}(\mathbf{V}_{\mathcal{D}} + \mathbf{Q})$, and hence:

$$\sigma'_{\mathcal{M}\mathcal{M}} = \frac{1}{3kT\Omega}\left(-\frac{m_{\mathcal{D}}}{m_{\mathcal{M}}}\right)^2 q_{\mathcal{M}}^2 \int_0^\infty \langle[\mathbf{V}_{\mathcal{D}}(t) + \mathbf{Q}(t)]\cdot[\mathbf{V}_{\mathcal{D}}(0) + \mathbf{Q}(0)]\rangle\, dt \tag{S17}$$

Since $\mathbf{Q}$ is non diffusive, $\sigma_{\mathcal{Q}\mathcal{Q}} \propto \int_0^\infty \langle \mathbf{Q}(t)\cdot\mathbf{Q}(0)\rangle\, dt = 0$. By Cauchy-Schwarz inequality, $\sigma_{\mathcal{D}\mathcal{Q}}$ also vanishes, hence $\sigma'_{\mathcal{M}\mathcal{M}} = \sigma_{\mathcal{M}\mathcal{M}}$ and $\sigma_{\mathcal{D}\mathcal{M}} = \sigma'_{\mathcal{D}\mathcal{M}}$. This concludes the proof.

[1] Albert P. Bartók, Mike C Payne, Risi Kondor, and Gábor Csányi, "Gaussian Approximation Potentials: The Accuracy of Quantum Mechanics, without the Electrons," Physical Review Letters **104**, 136403 (2010), arXiv:0910.1019.

[2] Volker L. Deringer, Albert P. Bartók, Noam Bernstein, David M. Wilkins, Michele Ceriotti, and Gábor Csányi, "Gaussian process regression for materials and molecules," Chemical Reviews **121**, 10073–10141 (2021), pMID: 34398616, https://doi.org/10.1021/acs.chemrev.1c00022.

[3] Peter Zaspel, Bing Huang, Helmut Harbrecht, and O. Anatole von Lilienfeld, "Boosting quantum machine learning models with a multilevel combination technique: Pople diagrams revisited," Journal of Chemical Theory and Computation **15**, 1546–1559 (2019).

[4] Félix Musil, Max Veit, Alexander Goscinski, Guillaume Fraux, Michael J Willatt, Markus Stricker, Till Junge, and Michele Ceriotti, "Efficient implementation of atom-density representations," The Journal of Chemical Physics **154** (2021).

[5] A Goscinski, VP Principe, G Fraux, S Kliavinek, BA Helfrecht, P Loche, M Ceriotti, and RK Cersonsky, "scikit-matter : A suite of generalisable machine learning methods born out of chemistry and materials science [version 2; peer review: 1 approved, 1 approved with reservations]," Open Research Europe **3** (2023), 10.12688/openreseurope.15789.2.

[6] Félix Musil, Michael J. Willatt, Mikhail A. Langovoy, and Michele Ceriotti, "Fast and Accurate Uncertainty Estimation in Chemical Machine Learning," Journal of Chemical Theory and Computation **15**, 906–915 (2019), publisher: American Chemical Society.

[7] Bernhard Schölkopf, Alexander Smola, and Klaus-Robert Müller, "Kernel principal component analysis," in *International conference on artificial neural networks* (Springer, 1997) pp. 583–588.

[8] Claudio Zeni, Andrea Anelli, Aldo Glielmo, and Kevin Rossi, "Exploring the robust extrapolation of high-dimensional machine learning potentials," Phys. Rev. B **105**, 165141 (2022).

[9] Sanggyu Chong, Federico Grasselli, Chiheb Ben Mahmoud, Joe D. Morrow, Volker L. Deringer, and Michele Ceriotti, "Robustness of local predictions in atomistic machine learning models," Journal of Chemical Theory and Computation **19**, 8020–8031 (2023), pMID: 37948446, https://doi.org/10.1021/acs.jctc.3c00704.

[10] Paolo Giannozzi, Stefano Baroni, Nicola Bonini, Matteo Calandra, Roberto Car, Carlo Cavazzoni, Davide Ceresoli, Guido L Chiarotti, Matteo Cococcioni, Ismaila Dabo, Andrea Dal Corso, Stefano de Gironcoli, Stefano Fabris, Guido Fratesi, Ralph Gebauer, Uwe Gerstmann, Christos Gougoussis, Anton Kokalj, Michele Lazzeri, Layla Martin-Samos, Nicola Marzari, Francesco Mauri, Riccardo Mazzarello, Stefano Paolini, Alfredo Pasquarello, Lorenzo Paulatto, Carlo Sbraccia, Sandro Scandolo, Gabriele Sclauzero, Ari P Seitsonen, Alexander Smogunov, Paolo Umari, and Renata M Wentzcovitch, "QUANTUM ESPRESSO: a modular and open-source software project for quantum simulations of materials," J. Phys. Cond. Matt. **21**, 395502–395519 (2009).

[11] P. Giannozzi, O. Andreussi, T. Brumme, O. Bunau, M. Buongiorno Nardelli, M. Calandra, R. Car, C. Cavazzoni, D. Ceresoli, M. Cococcioni, N. Colonna, I. Carnimeo, A. Dal Corso, S. de Gironcoli, P. Delugas, R. A. DiStasio, A. Ferretti, A. Floris, G. Fratesi, G. Fugallo, R. Gebauer, U. Gerstmann, F. Giustino, T. Gorni, J. Jia, M. Kawamura, H.-Y. Ko, A. Kokalj, E. Küçükbenli, M. Lazzeri, M. Marsili, N. Marzari, F. Mauri, N. L. Nguyen, H.-V. Nguyen, A. Otero-de-la Roza, L. Paulatto, S. Poncé, D. Rocca, R. Sabatini, B. Santra, M. Schlipf, A. P. Seitsonen, A. Smogunov, I. Timrov, T. Thonhauser, P. Umari, N. Vast, X. Wu, and S. Baroni, "Advanced capabilities for materials modelling with Quantum ESPRESSO," Journal of Physics: Condensed Matter **29**, 465901 (2017), publisher: IOP Publishing.

[12] Paolo Giannozzi, Oscar Baseggio, Pietro Bonfà, Davide Brunato, Roberto Car, Ivan Carnimeo, Carlo Cavazzoni, Stefano de Gironcoli, Pietro Delugas, Fabrizio Ferrari Ruffino, Andrea Ferretti, Nicola Marzari, Iurii Timrov, Andrea Urru, and Stefano Baroni, "Quantum ESPRESSO toward the exascale," The Journal of Chemical Physics **152**, 154105 (2020).

[13] Ivan Carnimeo, Fabio Affinito, Stefano Baroni, Oscar Baseggio, Laura Bellentani, Riccardo Bertossa, Pietro Davide Delugas, Fabrizio Ferrari Ruffino, Sergio Orlandini, Filippo Spiga, and Paolo Giannozzi, "Quantum espresso: One further step toward the exascale," Journal of Chemical Theory and Computation **19**, 6992–7006 (2023).

[14] John P. Perdew, Adrienn Ruzsinszky, Gábor I. Csonka, Oleg A. Vydrov, Gustavo E. Scuseria, Lucian A. Constantin, Xiaolan Zhou, and Kieron Burke, "Restoring the density-gradient expansion for exchange in solids and surfaces," Phys. Rev. Lett. **100**, 136406 (2008).

[15] G. Kresse and J. Hafner, "Ab initio molecular dynamics for liquid metals," Phys. Rev. B **47**, 558–561 (1993).

[16] G. Kresse and J. Furthmüller, "Efficiency of ab-initio total energy calculations for metals and semiconductors using a plane-wave basis set," Computational Materials Science **6**, 15–50 (1996).

[17] G. Kresse and J. Furthmüller, "Efficient iterative schemes for ab initio total-energy calculations using a plane-wave basis set," Phys. Rev. B **54**, 11169–11186 (1996).

[18] P. E. Blöchl, "Projector augmented-wave method," Phys. Rev. B **50**, 17953–17979 (1994).

[19] G. Kresse and D. Joubert, "From ultrasoft pseudopotentials to the projector augmented-wave method," Phys. Rev. B **59**, 1758–1775 (1999).

[20] R. Car and M. Parrinello, "Unified Approach for Molecular Dynamics and Density-Functional Theory," Physical Review Letters **55**, 2471–2474 (1985), publisher: American Physical Society.

[21] Shuichi Nosé, "A unified formulation of the constant temperature molecular dynamics methods," The Journal of Chemical Physics **81**, 511–519 (1984).

[22] Andrew M. Rappe, Karin M. Rabe, Efthimios Kaxiras, and J. D. Joannopoulos, "Optimized pseudopotentials," Phys. Rev. B **41**, 1227–1230 (1990).

[23] Aris Marcolongo and Nicola Marzari, "Ionic correlations and failure of nernst-einstein relation in solid-state electrolytes," Phys. Rev. Mater. **1**, 025402 (2017).

[24] Aris Marcolongo, Paolo Umari, and Stefano Baroni, "Microscopic theory and quantum simulation of atomic heat transport," Nature Physics **12**, 80–84 (2016).

[25] Federico Grasselli and Stefano Baroni, "Invariance principles in the theory and computation of transport coefficients," European Physical Journal B **94**, 160 (2021), 2105.02137.
